# Supplementary material for: Dynamic partitioning of branched-chain amino acids-derived nitrogen supports renal cancer progression
Source: Nat Commun. 2022 Dec 20;13:7830. doi: 10.1038/s41467-022-35036-4 (PMC9767928; doi:10.1038/s41467-022-35036-4)
Supplement: Supplementary file 1 — Supplementary Information [file 41467_2022_35036_MOESM1_ESM.pdf]

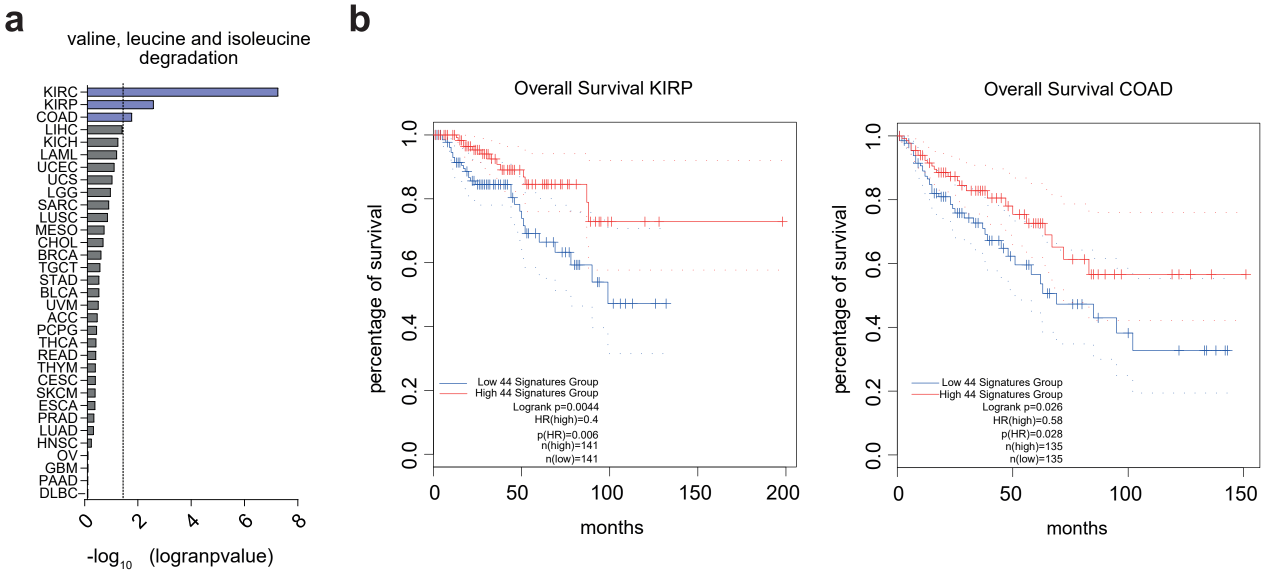

Supplementary Fig.1. The expression of the BCAA degradation pathway correlates with TCGA patients' survival. a, Bar plot showing the significance of the correlation between BCAA catabolism expression and patients' survival expressed as log10(p-value) for all TCGA tumors calculated using GEPIA. **b**, Overall survival of KIRP and COAD patients obtained through online tool GEPIA, based on gene expression of KEGG 'valine, leucine and isoleucine degradation' signature. For both panel a-b the cut-off used for high/low groups was 50% and p-value calculated as logrank(p-value) using Mantel–Cox test. Dotted line refers to the survival with a confidence interval (CI) of 95%. n=number of samples compared; HR=hazard ratio based on the Cox PH model. KIRP= renal papillary carcinoma, COAD= colorectal tumors.

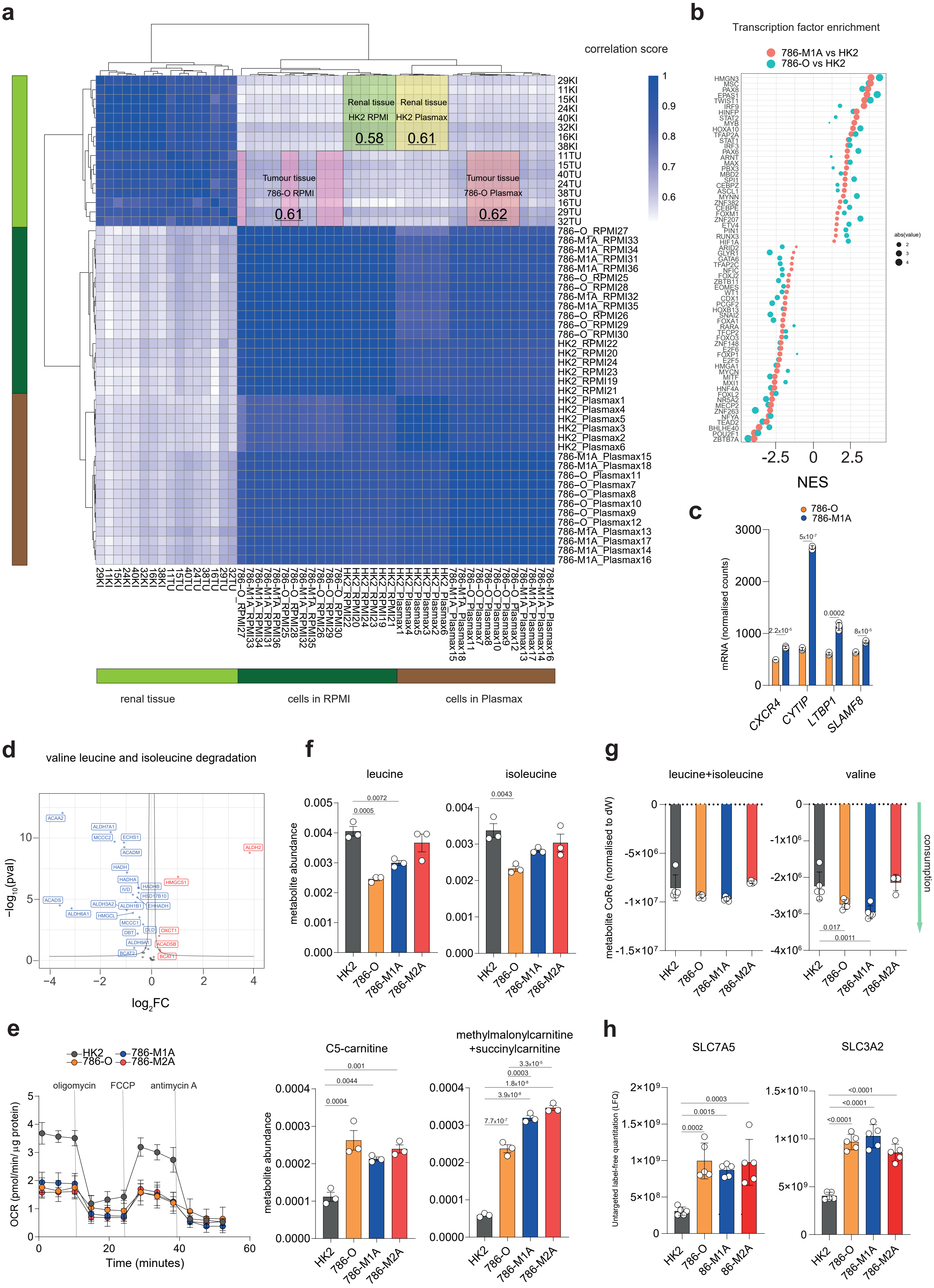

a
correlation score
Renal tissue HK2 RPMI 0.58
Renal tissue HK2 Plasmax 0.61
Tumour tissue 786-O RPMI 0.61
Tumour tissue 786-O Plasmax 0.62
renal tissue
cells in RPMI
cells in Plasmax
b
Transcription factor enrichment
786-M1A vs HK2
786-O vs HK2
NES
c
786-O
786-M1A
mRNA (normalised counts)
CXCR4
CYTIP
LTBP1
SLAMF8
d
valine leucine and isoleucine degradation
log2FC
−log10(pval)
e
HK2
786-O
786-M1A
786-M2A
oligomycin
FCCP
antimycin A
OCR (pmol/min/μg protein)
Time (minutes)
C5-carnitine
methylmalonylcarnitine +succinylcarnitine
metabolite abundance
f
leucine
isoleucine
metabolite abundance
g
leucine+isoleucine
valine
metabolite CoRe (normalised to dW)
consumption
h
SLC7A5
SLC3A2
Untargeted label-free quantitation (LFQ)

Supplementary Fig.2. ccRCC cells cultured in Plasmax resemble the metabolic and transcriptional profile of renal tumors. **a**, Heatmap showing the Pearson's correlation score between the metabolic profile of cells cultured in RPMI or Plasmax vs renal tumors (TU) and renal tissues, (KI). The average Pearson's correlation score value is indicated. **b**, Dot plot of the Transcription factor (TF) score comparing 786-O vs HK2 (green), 786-M1A vs HK2 (orange), ranked by significance from RNA-seq data. The dimension of the dots is based on the -log10(adj-p-value). NES= normalized enrichment score. TF enrichment scores were computed with the Viper R package, using minimum regular size of 5. The regions were obtained from the Dorothea R package, using interactions of confidence class A, B, C and D. **c**, Bar plot showing the normalized count of the mRNA for the indicated genes from RNA-seq. Significance was calculated using two-tailed unpaired t-test on log_2-transformed counts. Values are shown as mean ±SD from 3 independent cultures. **d**, Volcano plot showing the differential expression of proteins present in KEGG 'Valine leucine and isoleucine degradation' signature comparing 786-O vs HK2 cells. FC=fold change; red=upregulated blue=downregulated genes. **e**, Oxygen consumption rate (OCR) normalized for protein content/well of the renal cells in Plasmax. Values represent the mean of 4 independent cultures ±SD. **f**, Abundance of key metabolites from BCAA catabolism in renal cells from metabolomics. Data, normalized to total ion count, represent the mean of 3 independent experiments (N=3) ±S.E.M. **g**, Consumption/release of the indicated metabolites from Plasmax normalized to dry weight generation at t=24 (dW). Data are shown as mean of 5 independent cultures+ SD. **h**, Expression of the indicated proteins measured through labelled-free quantification (LFQ) proteomics in renal cells. Data are displayed as mean of 5 independent cultures ±SD. Significance was calculated using one-way ANOVA with multiple comparisons for panels f, g, h where each group was compared with HK2 (except for methylmalonylcarnitine in panel f). CXCR4=C-X-C Motif Chemokine Receptor 4; CYTIP=Cytohesin 1 Interacting Protein; LTBP1=Latent Transforming Growth Factor Beta Binding Protein 1; SLAMF8=SLAM Family Member 8; SLC7A5=Solute Carrier Family 7 Member 5; SLC3A2=Solute Carrier Family 3 Member 2.

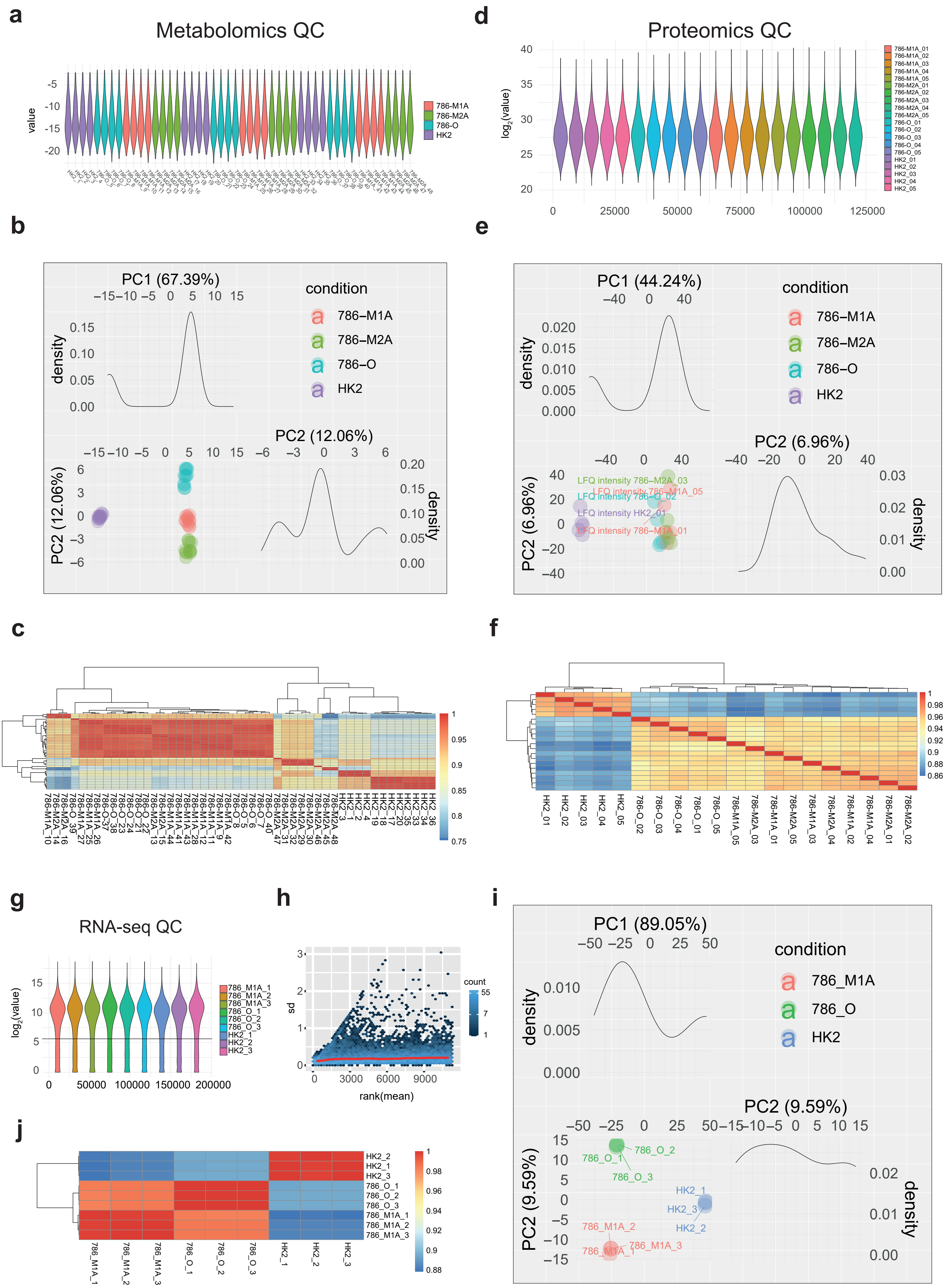
a
Metabolomics QC
value
786-M1A
786-M2A
786-O
HK2
b
PC1 (67.39%)
PC2 (12.06%)
density
condition
786–M1A
786–M2A
786–O
HK2
c
d
Proteomics QC
log2(value)
e
PC1 (44.24%)
PC2 (6.96%)
density
condition
786–M1A
786–M2A
786–O
HK2
LFQ intensity 786–M2A_03
LFQ intensity 786–M1A_05
LFQ intensity 786–O_02
LFQ intensity HK2_01
LFQ intensity 786–M1A_01
f
g
RNA-seq QC
log2(value)
786_M1A_1
786_M1A_2
786_M1A_3
786_O_1
786_O_2
786_O_3
HK2_1
HK2_2
HK2_3
h
sd
count
rank(mean)
i
PC1 (89.05%)
PC2 (9.59%)
density
condition
786_M1A
786_O
HK2
786_O_1
786_O_2
786_O_3
HK2_1
HK2_2
HK2_3
786_M1A_1
786_M1A_2
786_M1A_3
j

Supplementary Fig.3. Preliminary analyses (QC) of the multi-omic datasets generated from renal cells cultured in Plasmax.

a, Violin plots representing the normalized log_2 intensity distribution of the intracellular extract samples from the metabolomic dataset. **b**, Scatter plot of the first two principal components of the normalized log_2 intensities, with density curve of the first two components from the metabolomic dataset. **c**, Correlation heatmap of the metabolomic samples based on normalized log_2 intensities Pearson correlations. **d**, Violin plots representing the normalized log_2 intensity distributions of samples from the proteomic dataset. **e,** Scatter plot of the first two principal components of the normalized log_2 intensities, with density curve of the first two components from the proteomic dataset. **f**, Correlation heatmap of samples based on normalized log_2 intensities Pearson correlations from the proteomic dataset. **g**, Violin plots representing the log_2 count distribution of the RNA-seq samples. The horizontal line represents the minimum log_2(count) cutoff for low count filtering. **h,** Mean/sd plot of RNA counts after VSN normalization. **i**, Scatter plot of the first two principal components of the normalized count PCA, with density curve of the first two components relative to the RNA-seq samples. **j**, Correlation heatmap of RNA-seq samples based on normalized counts Pearson correlations. All datasets were generated from the indicated cells cultured in Plasmax.

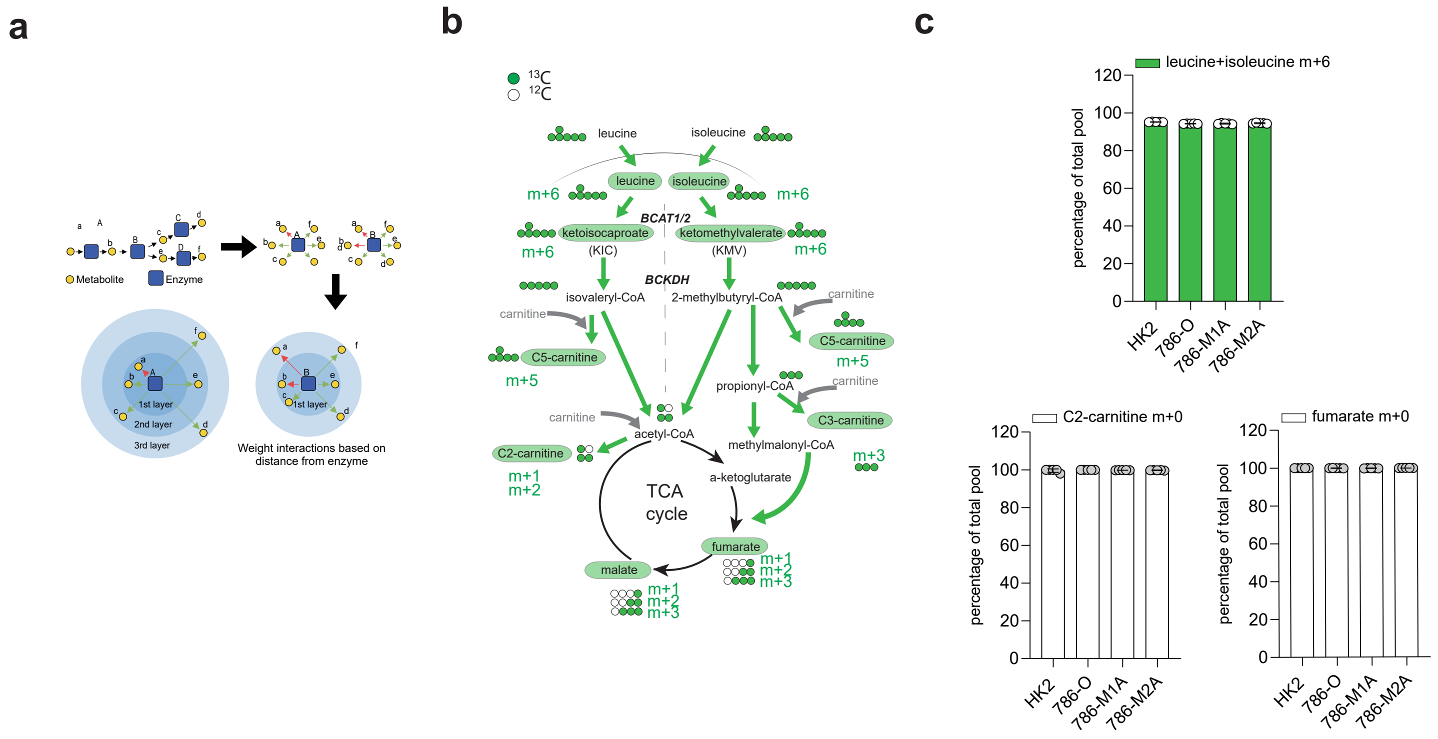

Supplementary Fig.4. BCAA catabolism does not provide carbons for the TCA cycle in ccRCC. **a**, Schematics showing how ocEAn computes the footprint for metabolic enzymes. **b**, Diagram of the labelling pattern originating from ^{13}C leucine+isoleucine catabolism. The green circles indicate ^{13}C, white circles represent unlabeled carbons. Measured metabolites through LC-MS are indicated in green circles. BCAT1/2= Branched Chain Amino Acid Transaminase 1/2; BCKDH = Branched Chain Keto Acid Dehydrogenase complex; KIC= ketoisocaproate. KMV=ketomethylvalearate. **c**, Proportion of total pool of the indicated labelled metabolites originating from ^{13}C leucine+isoleucine after 43h in the indicated cell lines. Data represent the mean of 5 independent cultures ±SD.

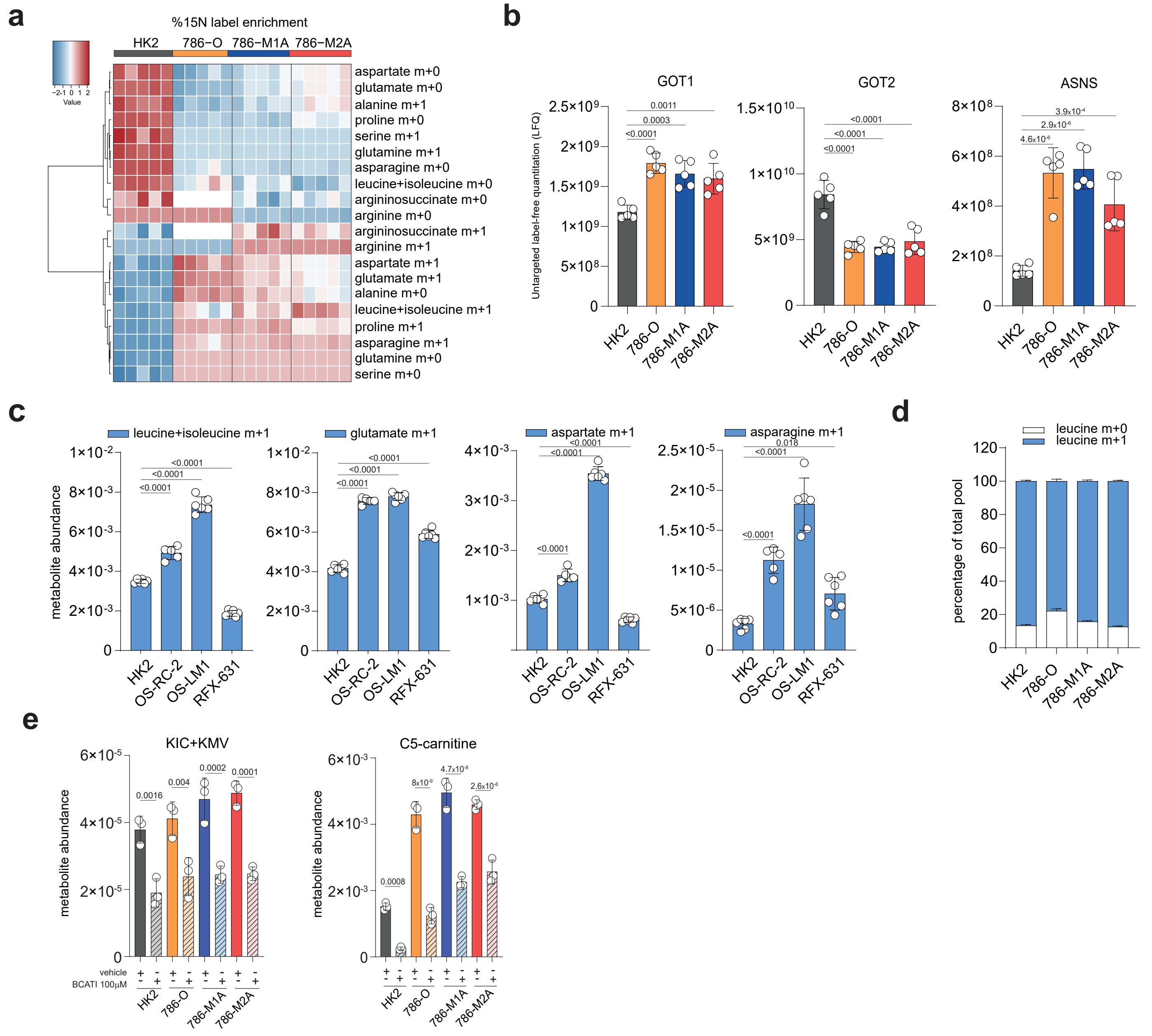

Supplementary Fig.5. BCAT transamination supplies an additional aspartate pool in multiple ccRCC cell lines. a, Heatmap showing the relative percentage of labelled metabolites m+1 on the nitrogen derived from ^{15}N Leucine, from the experiment indicated in Figure 4A. **b**, Labelled-free quantification (LFQ) of the indicated proteins based on proteomics dataset generated after culturing cells in Plasmax. Data are shown as mean of 5 independent cultures ±SD. Significance was calculated using one-way ANOVA where each group was compared with HK2. **c**, Abundances of labelled leucine m+1, glutamate m+1, aspartate m+1 and asparagine m+1 originating from ^{15}N leucine+isoleucine in Plasmax after 27h in additional ccRCC cells lines. Data are normalized to total ion count and represent the mean of 6 independent cultures ±SD. p-values were calculated using one-way ANOVA where each group was compared with HK2. **d**, Proportion of total pool of intracellular leucine after incubation of the cells with ^{15}N leucine EBSS+FBS 2.5% for 24h. Data represent the mean of 6 independent cultures ±SD. **e**, Intracellular abundance of the indicated metabolites after treatment with BCATI 100µM in Plasmax for 22h. Values are normalized to total ion count and expressed as the mean of 3 independent cultures ±SD. p-values were calculated using one-way ANOVA with multiple comparisons and indicated in the graph for the comparisons treated vs vehicle for all biological groups.

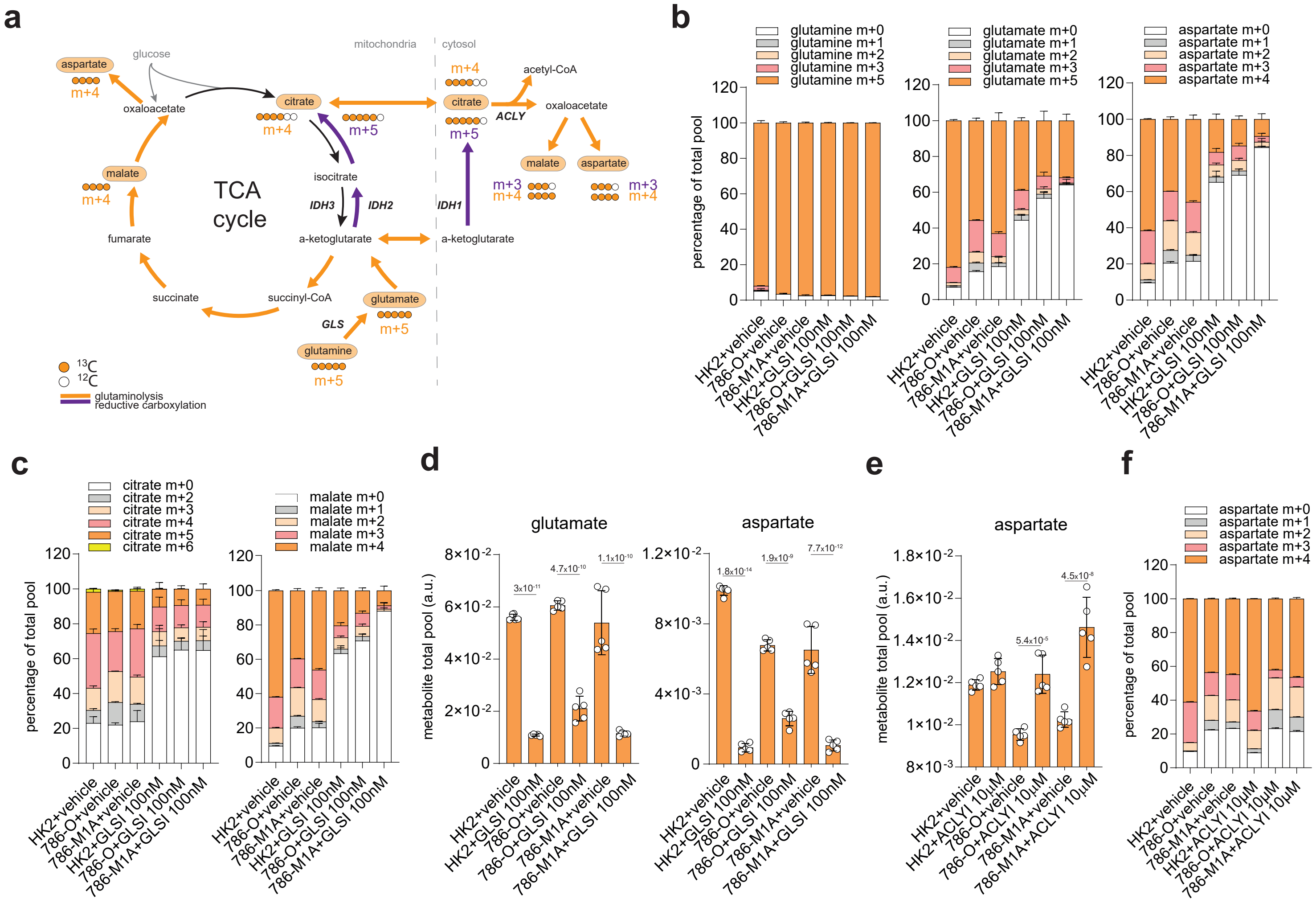

Supplementary Fig.6. Glutaminolysis contribution to the carbon backbone of aspartate and glutamate in ccRCC cells.

a, Diagram of the labelling pattern originating from ^{13}C glutamine catabolism. The orange circles indicate ^{13}C, white circles represent unlabeled carbons. Measured metabolites through LC-MS are indicated in orange. Purple arrows and isotopologues indicate labelling patterns derived from glutamine reductive carboxylation.; ACLY= ATP Citrate Lyase; IDH= isocitrate dehydrogenase; GLS= glutaminase. **b-c**, Proportion of total pool of the indicated labelled metabolites originating from ^{13}C glutamine after 22h in the indicated cell lines treated with vehicle GLSI (CB-839, 100nM) in Plasmax for 23h. Data represent the mean of 5 independent cultures ±SD. **d**, Intracellular abundance of the indicated metabolites after treatment with GLSI (CB-839) in Plasmax for 23h. Values are normalized to total ion count and expressed as the mean of 5 independent cultures ±SD. p-values were calculated using one-way ANOVA with multiple comparisons and indicated in the graph for the comparisons treated vs vehicle for all biological groups **e**, Intracellular abundance of aspartate after treatment with ACLYI (BMS-303141, 10µM) in Plasmax for 8h. Values are normalized to total ion count and expressed as the mean of 5 independent cultures ±SD. p-values were calculated using one-way ANOVA with multiple comparisons and indicated in the graph for the comparisons treated vs vehicle for all biological groups. **f,** Proportion of total pool of aspartate originating from ^{13}C glutamine after 8h in the indicated cell lines treated with ACLYI. Data represent the mean of 5 independent cultures ±SD.

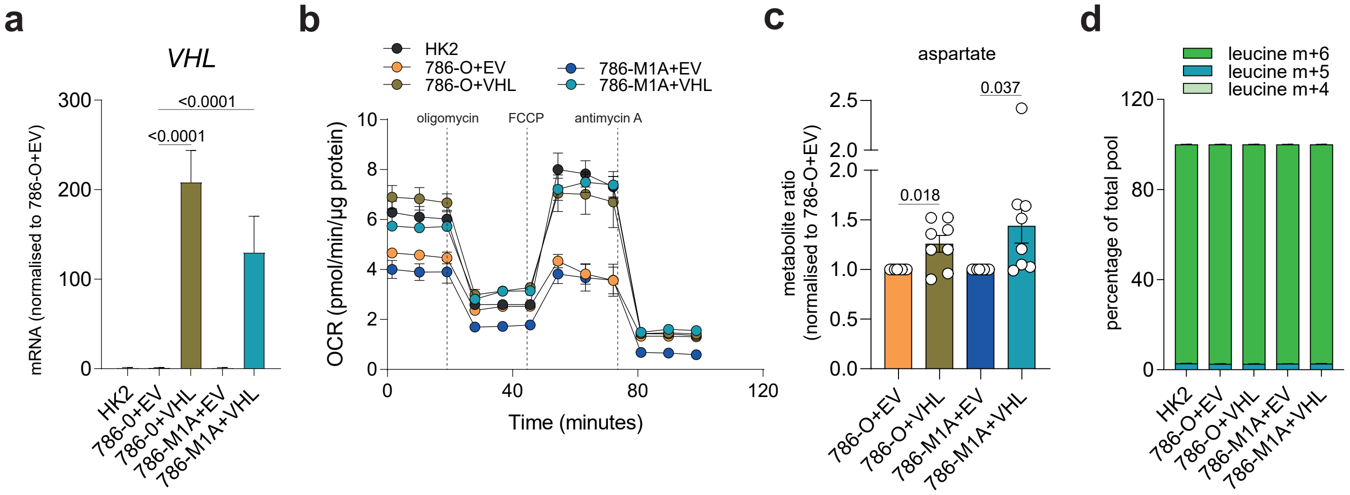

Supplementary Fig.7. VHL reconstitution restores mitochondrial function and aspartate level in ccRCC cells. a, mRNA levels of *VHL* in the indicated cell lines grown in RPMI through qPCR. *TBP* was used as endogenous control. Values represent relative quantification (RQ) ± error calculated using Expression suite software (Applied biosystem) calculated using SD algorithm. p-values were calculated through Expression suite software. N=3 independent experiments. **b**, Cellular respiration of the indicated cell line cultured in RPMI after VHL re-expression using Sea Horse Extracellular flux analyzer XF24. OCR=oxygen consumption rate normalized for protein content/well. Values are represented as the mean of 3 independent experiments ±S.E.M. (N=3). **c**, Ratio of the intracellular abundance of aspartate in cells grown in RPMI expressing VHL compared to EV. Data were normalized to total ion count and represent the mean of 8 independent experiments (N=8) ±S.E.M. p-values were calculated using a two-tailed one sample t-test against the theoretical mean of 1 (786-O+EV=1 vs 786-O+VHL and 786-M1A+EV=1 vs 786-M1A+VHL). **d**, Proportion of total pool of the intracellular leucine. Cells were grown for 24h in RPMI+^{13}C leucine. Data represent the mean of 5 independent cultures ±SD.

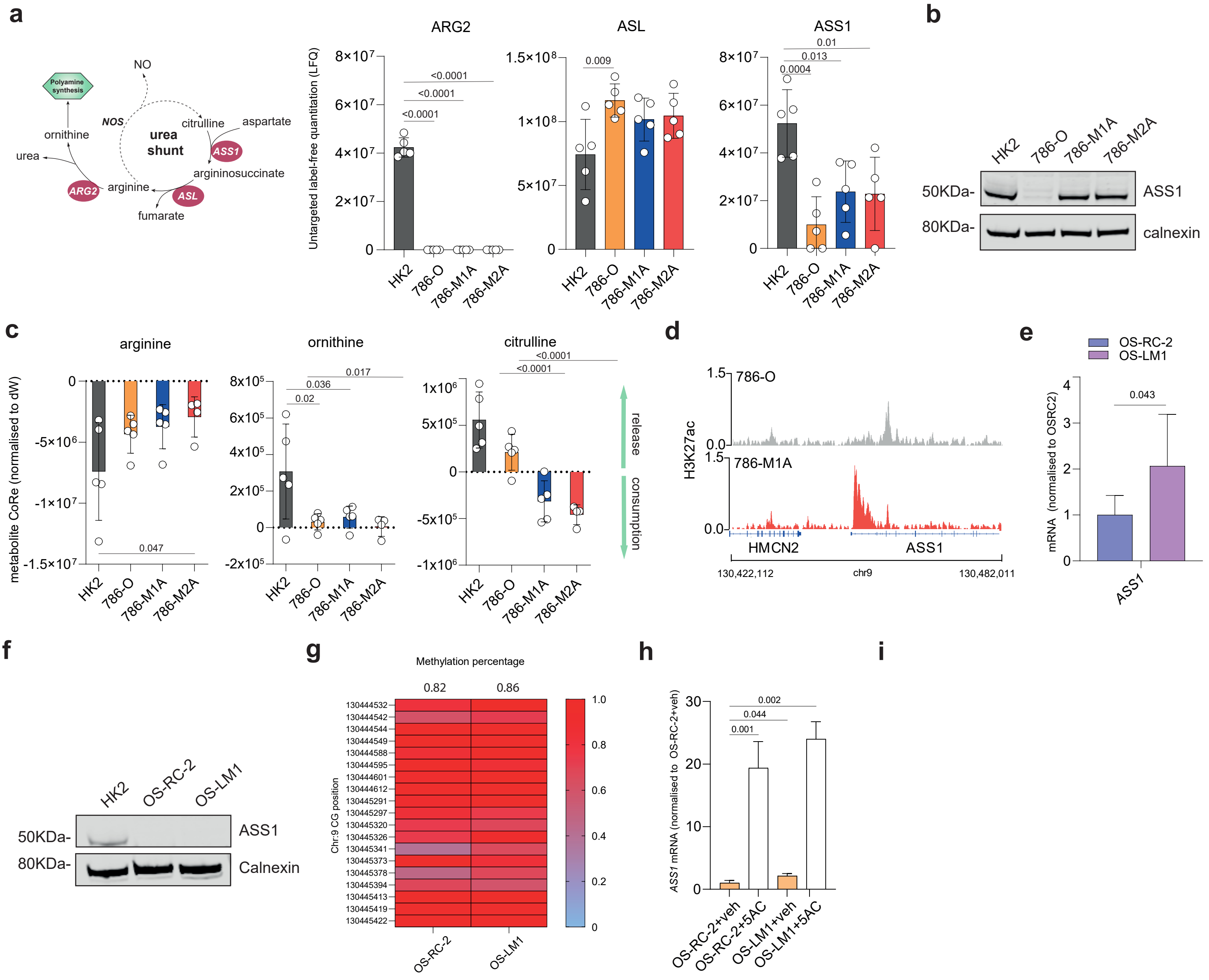

Supplementary Fig.8. *ASS1* regulation in metastatic 786-M1A and OS-LM1 cells. a, Schematics of the urea shunt in renal cells (left) and Arginase 2 (ARG2), argininosuccinate synthase (ASS1) and argininosuccinate lyase (ASL) protein expression (right) measured through labelled-free quantification (LFQ) from the proteomic dataset in the indicated cell lines. Data are shown as mean of 5 independent cultures ±SD. Significance was calculated using one-way ANOVA where each group was compared with HK2. **b**, Western blot of the ASS1 levels in cells stably cultured in Plasmax. Calnexin was used as an endogenous control. **c**, Consumption/release of arginine, ornithine and citrulline from medium normalized to dry weight generation at t=24 (dW) in the indicated cell lines. Data represent the mean of 5 independent cultures ±SD. p-values were calculated using one-way ANOVA with multiple comparisons where each group was compared with HK2. **d**, Graphical visualization of the acetylation H3k27ac peaks for the genomic region around ASS1 gene in 786-O and 786-M1A generated using IGV software. Data were previously generated (Rodrigues et al.2018) from cells grown in RPMI. **e**, mRNA levels of *ASS1* in OS-RC-2 and OS-LM1 through qPCR. *TBP* was used as endogenous control. Values represent relative quantification (RQ) ± error. Errors, based on SD algorithm and p-value were calculated using Expression suite software (Applied biosystem) N=4 independent experiments. **f**, Western blot of ASS1 protein level in cells indicated stably cultured in Plasmax. Calnexin was used as an endogenous control. **g**, Heatmap showing the methylation level (B-value) of the indicated CG from a CpG island overlapping with *ASS1* TSS. Values are presented as the mean of two independent experiments. OS-RC-2 and OS-LM1 were grown in RPMI. **h**, mRNA levels of *ASS1* in OS-RC-2 and OS-LM1 treated for 72h with either vehicle or 5AC 200nM measured through qPCR. *TBP* was used as endogenous control. Values represent relative quantification (RQ) ± error. Errors, based on SD algorithm and p-value were calculated using Expression suite software (Applied biosystem). N=3 independent experiments.

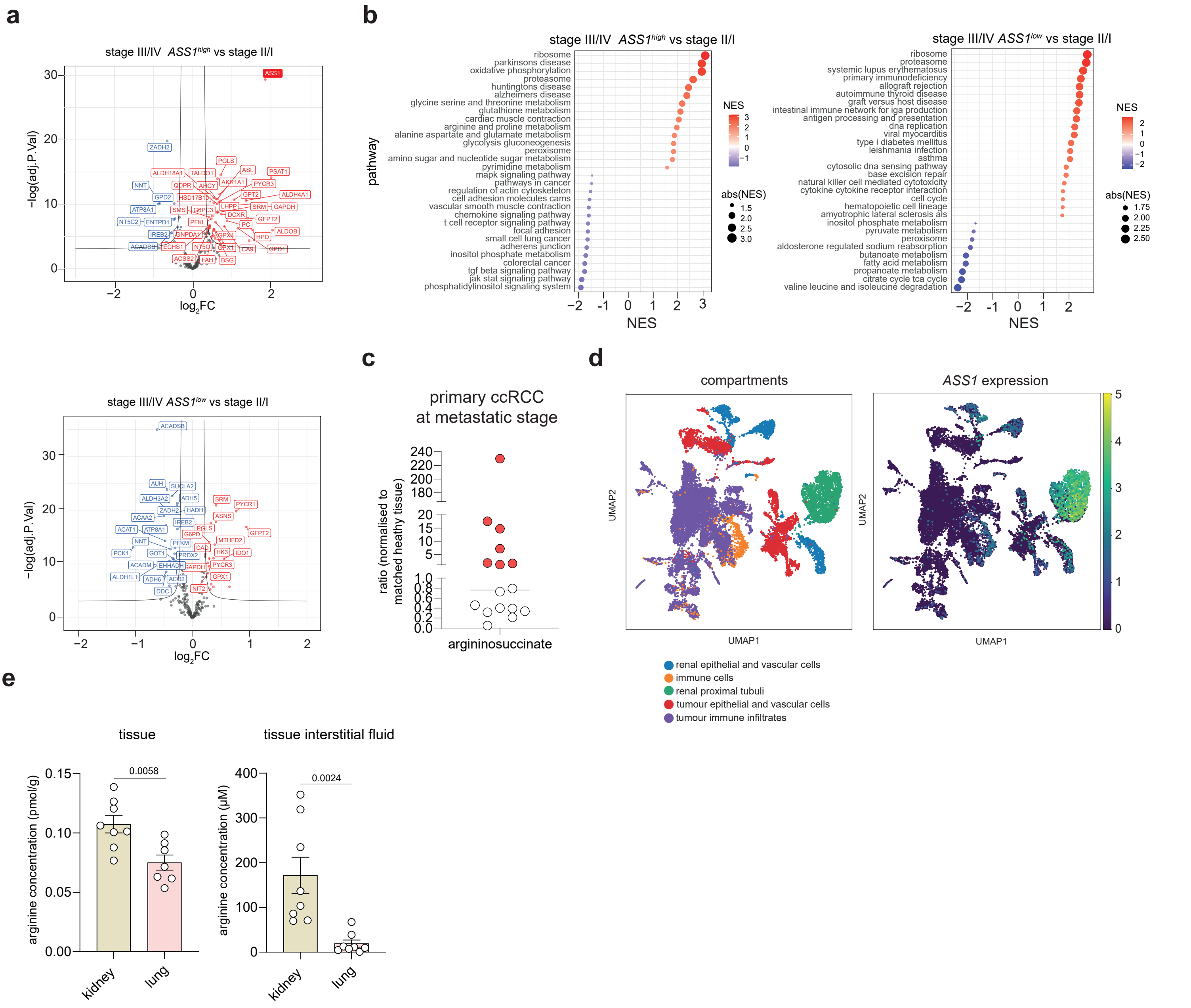

Supplementary Fig.9. *ASS1* expression in advanced ccRCC tumors. **a**.Volcano plot of the metabolic genes differentially expressed in a cluster of TCGA KIRC advanced tumors (Stage III+IV) where *ASS1* expression is high (*ASS1*^{high}) or low (*ASS1*^{low}) compared to tumors from Stage I+II. Fold change is expressed as $\log_2$FC. Y axis represents -log10(p-value). **b**, GSEA of the pathways expressed in a cluster of TCGA KIRC advanced tumors (Stage III+IV) where *ASS1* expression is higher (*ASS1*^{high}) or lower (*ASS1*^{low}) compared to tumors from Stage I+II. NES=normalized enrichment score. **c**, Ratio of the argininosuccinate measured through LC-MS in a cohort of ccRCC patients' primary tumors that were metastatic at the time of diagnosis, normalized to healthy matched tissue. Red dots indicate ratio values>1, while white dots <1. **d**, Expression of *ASS1* based on single-cell RNA-seq from three ccRCC patients including the cell populations of origin (compartments, as defined in the original publication) displayed on the first two components of a UMAP dimensionality reduction plot. **e**, Arginine levels in the tissue or the interstitial fluid from mouse renal and lung tissues. Data represent the mean of 8 mice ± S.E.M. p-values were calculated using unpaired t-test.

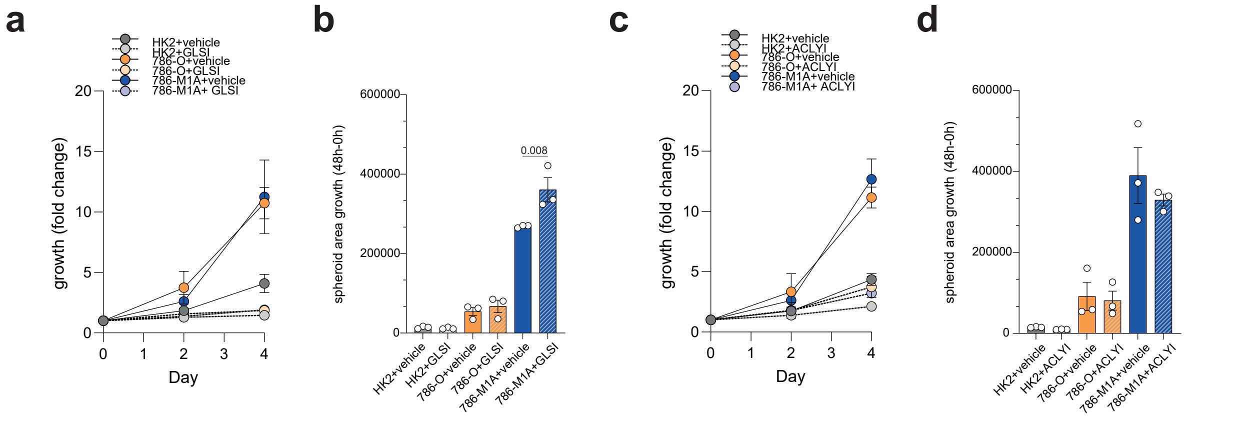

Supplementary Fig.10. Glutaminolysis does not support the invasion of ccRCC cells *in vitro.*
a-c, Proliferation rate of the indicated cell lines in the presence of 1µM of GLSI (CB-839) or 10µM ACLYI (BMS-303141) in Plasmax. Data represent the mean of 3 independent experiments ± S.E.M. Values represent fold change increase of growth relative to day 0. **b-d,** Quantification of the increase in area of spheroids included in collagen I matrix (time 48h-0h). N=3 independent experiments± S.E.M. Statistical significance was calculated using one-way ANOVA with multiple comparisons and indicated in the graph for the comparison treated vs vehicle for all biological groups.
