# Supplementary material for: Dynamic partitioning of branched-chain amino acids-derived nitrogen supports renal cancer progression
Source: Nat Commun. 2022 Dec 20;13:7830. doi: 10.1038/s41467-022-35036-4 (PMC9767928; doi:10.1038/s41467-022-35036-4)
Supplement: Supplementary file 3 — Description of Additional Supplementary Files [file 41467_2022_35036_MOESM3_ESM.pdf]

Description of Additional Supplementary Files

File Name: Supplementary Data 1

Description: **Metabolic reaction network for ocEAn.**

Reaction table from the redHuman metabolic network in Simple Interaction Format. Each row represents the directed interaction between a source (metabolite or enzyme) and a target (metabolite or enzyme. Metabolite are identified by KEGG ID when applicable and genes are identified with HUGO symbols.
