# Supplementary material for: Dynamic partitioning of branched-chain amino acids-derived nitrogen supports renal cancer progression
Source: Nat Commun. 2022 Dec 20;13:7830. doi: 10.1038/s41467-022-35036-4 (PMC9767928; doi:10.1038/s41467-022-35036-4)
Supplement: Supplementary file 5 — Reporting Summary [file 41467_2022_35036_MOESM5_ESM.pdf]

nature portfolio

Corresponding author(s): Christian Frezza

Last updated by author(s): 18/10/22

Reporting Summary

Nature Portfolio wishes to improve the reproducibility of the work that we publish. This form provides structure for consistency and transparency in reporting. For further information on Nature Portfolio policies, see our Editorial Policies and the Editorial Policy Checklist.

Statistics

For all statistical analyses, confirm that the following items are present in the figure legend, table legend, main text, or Methods section.

n/a	Confirmed	
☐	☒	The exact sample size (*n*) for each experimental group/condition, given as a discrete number and unit of measurement
☐	☒	A statement on whether measurements were taken from distinct samples or whether the same sample was measured repeatedly
☐	☒	The statistical test(s) used AND whether they are one- or two-sided<br>*Only common tests should be described solely by name; describe more complex techniques in the Methods section.*
☒	☐	A description of all covariates tested
☐	☒	A description of any assumptions or corrections, such as tests of normality and adjustment for multiple comparisons
☐	☒	A full description of the statistical parameters including central tendency (e.g. means) or other basic estimates (e.g. regression coefficient) AND variation (e.g. standard deviation) or associated estimates of uncertainty (e.g. confidence intervals)
☐	☒	For null hypothesis testing, the test statistic (e.g. *F*, *t*, *r*) with confidence intervals, effect sizes, degrees of freedom and *P* value noted<br>*Give P values as exact values whenever suitable.*
☒	☐	For Bayesian analysis, information on the choice of priors and Markov chain Monte Carlo settings
☒	☐	For hierarchical and complex designs, identification of the appropriate level for tests and full reporting of outcomes
☐	☒	Estimates of effect sizes (e.g. Cohen's *d*, Pearson's *r*), indicating how they were calculated

Our web collection on statistics for biologists contains articles on many of the points above.

Software and code

Policy information about availability of computer code

Data collection

All data and script used for the analysis of metabolomic, proteomic and RNA dataset used are available at: DOI: https://zenodo.org/badge/latestdoi/395034170

Data analysis

ocEAn package is available at: DOI: https://zenodo.org/badge/latestdoi/395034170

Other software used are:
Graphpad Prism 9
TrimGalore v0.4.4
Bismark (v0.22.1)
FastQC (v0.11.4)
Expression Suite v1.1 (Thermo Scientific)
Image Studio Lite (v5.2)
StepOne Software v2.3
Wave v2.6 (Agilent)
Integrative Genomics Viewer (IGV)- Broad Institute v2.7
RStudio-2022.07.2-576
Fiji software or ImageJ 1.52p-Java 8
MaxQuant software package 1.6

For manuscripts utilizing custom algorithms or software that are central to the research but not yet described in published literature, software must be made available to editors and reviewers. We strongly encourage code deposition in a community repository (e.g. GitHub). See the Nature Portfolio guidelines for submitting code & software for further information.

Data

Policy information about availability of data

All manuscripts must include a data availability statement. This statement should provide the following information, where applicable:

- Accession codes, unique identifiers, or web links for publicly available datasets
- A description of any restrictions on data availability
- For clinical datasets or third party data, please ensure that the statement adheres to our policy

The manuscript includes a Data availability section in the Methods.
RNA-seq and proteomic raw files are available at: DOI: https://zenodo.org/badge/latestdoi/395034170.
Metabolomic raw files are available at: https://www.metabolomicsworkbench.org/data/DRCCMetadata.php?Mode=Project&ProjectID=PR001418
Additional metabolomic experiments will be available on MetaboLights (curation on going).
All source data used to generate graphs in the manuscript are available in the Source Data File. Western Blot uncropped images are available in the Source Data File.

Field-specific reporting

Please select the one below that is the best fit for your research. If you are not sure, read the appropriate sections before making your selection.

[x] Life sciences [ ] Behavioural & social sciences [ ] Ecological, evolutionary & environmental sciences

For a reference copy of the document with all sections, see nature.com/documents/nr-reporting-summary-flat.pdf

Life sciences study design

All studies must disclose on these points even when the disclosure is negative.

Sample size	No statistical method was used to predetermine sample size. The sample sized used in the study is similar to most existing works in the same field.
Data exclusions	No data were excluded from the analysis
Replication	The reproducibility of the experimental findings was supported by performing independent experiments (usually n=3) or by having several independent culture replicates (replicate wells/dishes) as reported in the figure legends. Furthermore, additional experiments were conducted in other relevant cell lines to validate the main findings of the study. Western blot experiments were repeated more than once or validated with other techniques (qPCR or multi-omic analysis).
Randomization	the metabolomic samples were run in a randomized order
Blinding	Metabolomic samples were run blindly. For other experiments, the experimental groups were not blinded.

Reporting for specific materials, systems and methods

We require information from authors about some types of materials, experimental systems and methods used in many studies. Here, indicate whether each material, system or method listed is relevant to your study. If you are not sure if a list item applies to your research, read the appropriate section before selecting a response.

Materials & experimental systems

n/a	Involved in the study	
[ ]	[x]	Antibodies
[ ]	[x]	Eukaryotic cell lines
[x]	[ ]	Palaeontology and archaeology
[ ]	[x]	Animals and other organisms
[ ]	[x]	Human research participants
[x]	[ ]	Clinical data
[x]	[ ]	Dual use research of concern

Methods

n/a	Involved in the study	
[x]	[ ]	ChIP-seq
[x]	[ ]	Flow cytometry
[x]	[ ]	MRI-based neuroimaging

Antibodies

Antibodies used	Calnexin antibody was purchased from Abcam (ab22595, used at 1:1000 dilution), ASS1 from Abcam (ab124465 used at 1:500 dilution). Secondary antibodies (conjugated with 680 or 800 nm fluorophores, IRDye® 800CW Goat anti-Mouse IgG cat. 926-32210; IRDye® 800CW Goat anti-Rabbit IgG cat. 926-32211; IRDye® 680LT Goat anti-Mouse IgG cat. 926-68020; IRDye® 680LT Goat anti-Rabbit IgG cat. 926-68021, all diluted 1:2000) were purchased from LI-COR. Human Vimentin was purchased from Cell signaling

	(#5741) and used at 1:100 dilution.
Validation	Validation and specificity of the antibodies used in the study is described on the technical datasheets available at the webpage of the vendor. The webpage includes also reference of the studies published where the antibody was used.<br>1. Calnexin (ab22595): https://www.abcam.com/calnexin-antibody-er-marker-ab22595.html, Datasheet and documents section;<br>2. ASS1 (ab124465): https://www.abcam.com/ass1-antibody-2b10-ab124465.html,Datasheet and documents section;<br>3. Vimentin (Cell Signaling #5741): https://www.cellsignal.co.uk/products/primary-antibodies/vimentin-d21h3-xp-rabbit-mab/5741<br>4. IRDye® 680LT Goat anti-Mouse IgG (H + L): https://www.licor.com/bio/reagents/irdye-680lt-goat-anti-mouse-igg-secondary-antibody;<br>5. IRDye® 680LT Goat anti-Rabbit IgG (H + L):https://www.licor.com/bio/reagents/irdye-680lt-goat-anti-rabbit-igg-secondary-antibody<br>6.IRDye® 800CW Goat anti-Mouse IgG Secondary Antibody:https://www.licor.com/bio/reagents/irdye-800cw-goat-anti-mouse-igg-secondary-antibody;<br>7.IRDye® 800CW Goat anti-Rabbit IgG Secondary Antibody:https://www.licor.com/bio/reagents/irdye-800cw-goat-anti-rabbit-igg-secondary-antibody.

Eukaryotic cell lines

Policy information about cell lines

Cell line source(s)	Human renal cancer cell lines; 786-O, 786-M1A, OS-RC2, OS-LM1, were obtained from J. Massagué (MSKCC, New York, USA) in 2014. 786-M1A and OS-LM1 cells are metastatic derivatives of 786-O and OS-RC2 cells, respectively (Vanharanta et al., Nat Med. (2013) PMID: 23223005). RFX-631 cells were obtained from the National Cancer Institute (US) while HK2 from Eamonn Maher's laboratory (University of Cambridge). HK2, 786-O cells are available on ATCC catalogue, OS-RC-2 on RIKEN BRC and RFX-631 from National Cancer Institute (US).
Authentication	Cells were authenticated using y short tandem repeat (STR) analysis.
Mycoplasma contamination	Cells were routinely tested for mycoplasma contamination
Commonly misidentified lines<br>(See ICLAC register)	At the time of the study, none of the cell lines used in this study were listed in the database of ICLAC.

Animals and other organisms

Policy information about studies involving animals; ARRIVE guidelines recommended for reporting animal research

Laboratory animals	NOD/SCID mice, female, 7 weeks old, purchased from Charles River Laboratories. The housing for the animal experimental work carried out in this study was controlled by the animal facility at the University of Cambridge. Chow diet ad libitum was used during the experiments.
Wild animals	wild animals were not used in the study
Field-collected samples	there are no field-collected samples in the study
Ethics oversight	All animal experiments were performed in accordance with protocols approved by the Home Office (UK) and the University of Cambridge ethics committee (PPL PFCB122AA)

Note that full information on the approval of the study protocol must also be provided in the manuscript.

Human research participants

Policy information about studies involving human research participants

Population characteristics	In the cohort of samples used for the analysis, 67% of the patients were males, 33% females. The average age of the patients was 60 years with a range spanning between 24-81 years.
Recruitment	Patients were requited at the time of presentation into the DIAMOND study seeking to identify biomarkers of urological disease. Following informed consent, tissue samples surplus to diagnosis and management were banked and stored in our centre.
Ethics oversight	'DISCOVERY AND ANALYSIS OF NOVEL BIOMARKERS IN UROLOGICAL DISEASES (DIAMOND STUDY)'<br>REC ref. 03/018 (UK Health and Research authority)

Note that full information on the approval of the study protocol must also be provided in the manuscript.
